# An Estimation Approach for the Effective Elastic Modulus of Lightweight Bulk Filling Material with Compressible Inclusions and Imperfect Interfaces

**DOI:** 10.3390/ma13163563

**Published:** 2020-08-12

**Authors:** Chengxuan Li, Jianguo Wang, Fakai Dou

**Affiliations:** 1 School of Mechanics and Civil Engineering, China University of Mining and Technology, Xuzhou 221116, China; lichengxuan@cumt.edu.cn; 2 State Key Laboratory for Geomechanics and Deep Underground Engineering, China University of Mining and Technology, Xuzhou 221116, China; doufakai@cumt.edu.cn

**Keywords:** lightweight material, mean field homogenization, expended polystyrene beads, imperfect interface, density, effective elastic modulus

## Abstract

In this study, an approach is developed to estimate the density and effective elastic modulus of a lightweight bulk filling material made up of expanded polystyrene (EPS) and cement-reinforced clay (matrix). First, a representative volume element (RVE) is composed of cell A (an EPS and matrix) and cell B (matrix only). Then, an elastic interface is introduced to describe the discontinuity of displacement at the interface between EPS beads and matrix. Third, an Eshelby compliance tensor is modified in cell A to include the effects of imperfect interface and the compressibility of EPS beads. Finally, the approach for the density and effective elastic modulus of the EPS beads mixed cement-reinforced clay is verified with experimental data. The compressibility ratio of lightweight clay is compared under different confining pressures and curing times. It is found that the imperfect interface has salient impacts on the effective elastic modulus with the increase of volume fraction of inclusions. The interface parameters (*α* and *β*) vary with curing time and confining pressure. At the same curing time, the parameter *α* is almost constant regardless of confining pressure but the parameter *β* changes with confining pressure. The compressibility ratio is smaller for longer curing time if the confining pressure is constant.

## 1. Introduction

**Figure 1:**
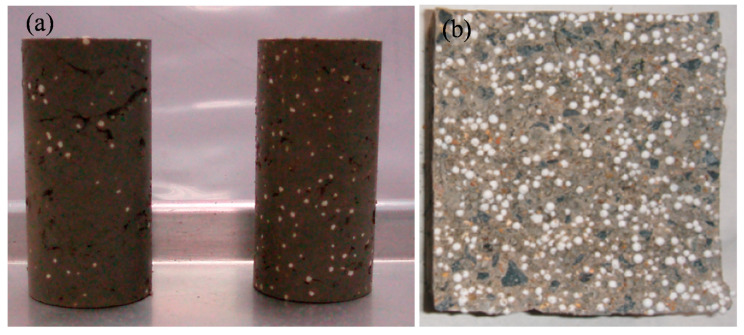
The lightweight bulk filling materials (**a**) lightweight clay [11]; (**b**) lightweight concrete [12].

Lightweight bulk filling material may be lightweight clay or lightweight concrete as shown in Figure 1. It has been widely used in construction, soft ground treatment and highway embankments. The expanded polystyrene (EPS) has some excellent physical and mechanical properties such as lightweight, excellent thermal and structural damping. Thus, the mechanical properties of lightweight materials have been investigated by experiments or modeling. For example, Solak et al. [1] proposed five different methods to quantify the segregation phenomenon in lightweight aggregate concretes (LWAC). Dixit et al. [2] developed a lightweight EPS cement composite material (LECC) with enhanced insulation and satisfactory compressive strength for structural applications. Gao et al. [3] studied the dynamic characteristics of expanded polystyrene (EPS) composite soil under complex stress paths. The characteristics of skeleton curve, dynamic shear modulus and damping ratio for EPS composite soil were analyzed. Herki et al. [4] studied the effects of the stabilized polystyrene (SPS) on the physical and mechanical properties of concrete. Wang et al. [5] explored the roles of expanded polystyrene (EPS) beads in the reinforcement of soft soil highway embankments through experimental study and numerical simulation. Miled et al. [6] tested five volume fractions of different expanded polystyrenes. They found that the EPS particle size affects the compressive strength of EPS concrete. Gao et al. [7] conducted a series of triaxial undrained creep tests to investigate the creep behaviors of EPS composite soil. Nguyen-Sy et al. [8] considered the orientations of hemp particles and imperfect particle-binding interfaces. They explored the effects of volume fraction of hemp particles on the overall thermal conductivity of hemp concrete. Further, the dynamic properties of ultra-high performance concrete (UHPC) were investigated with numerical simulations and artificial intelligence approach [9,10]. These investigations show that the physical and mechanical properties of lightweight bulk filling material vary with the volume fraction of inclusions and the interface properties. However, an approach is still unavailable to estimate the mechanical properties of lightweight bulk filling material.

An approach is necessary to analyze and evaluate the performance of lightweight materials and to design new lightweight materials [13,14,15,16]. The performance of a lightweight material can be measured by two key parameters: density and effective elastic modulus. The density of the lightweight material is very important to the structure sensitive to self-weight. Effective elastic modulus refers to the macroscopic elastic modulus of lightweight materials and is the primary consideration for new construction materials. Nguyen and Fatahi [17,18] presented a constitutive model for the fibre reinforced cement treated soil. Wang et al. [19] proposed a simple homogenization approach to calculate the deformation and strength parameters of composite soils which is composed of well-mixed soft and stiff soils. Chen et al. [20] presented a homogenization-based effective thermal conductivity model for unsaturated compacted bentonites (a kind of compacted soils). It is worth noting that most of the above studies have focused on the mechanical properties of fiber-reinforced soil or cement-reinforced clay. Their inclusions are incompressible. So far, the mechanical properties of composite materials with highly compressible inclusions have not been paid attentions yet.

The estimation of overall mechanical properties of composite materials with particulate inclusions is still a challenge [21]. Eshelby tensor-based homogenization schemes have been extensively studied when estimating the mechanical properties of composite material under elastic deformation [22,23,24]. In these schemes, the internal interaction among different inclusions is ignorable [25,26,27]. Such an approximation is questionable for high inclusion content where the interaction may be strong. For this interaction, Mori-Tanaka tensor-based schemes have been proposed and the effects of interface debonding on the macroscopic behavior of composite material were considered [28,29]. Imperfect interface refers to the discontinuity of displacement at the interface due to non-conformal deformation of the matrix and inclusions. The concept of imperfect interface was firstly proposed by Benceniste and Hashin [30,31,32]. This interaction among inclusions and the imperfect interface between inclusions and matrix are important factors affecting the effective properties of lightweight bulk filling material, thus should be carefully included.

In recent years, the study on imperfect interfaces between substrates and inclusions has gradually become a research hotspot [33,34,35,36]. For example, the homogenization schemes were proposed based on matched asymptotic method [37], periodic variational principle [38] and the two-scale asymptotics [39]. A boundary integral equation for imperfect interface was obtained with a plane electrostatic Green’s function [40,41]. However, most of the above studies focus on fiber-reinforced composites or cement-reinforced clay. The EPS beads mixed cement-reinforced clay have seldom been investigated so far.

This paper is to propose an approach to estimate the effective physical and mechanical properties of lightweight clay. The complex effects of different components and their interfaces are introduced into the estimation approach for the macroscopic mechanical behaviors. Particularly, the density and the effective elastic modulus of lightweight bulk filling material are estimated. The paper is organized as follows. In Section 2, a density estimation approach is proposed and verified by the density data of our EPS mixed cement reinforce clay. Section 3 proposes a linear elastic spring model to describe the imperfect interface between the EPS beads and the matrix. The effective stiffness coefficient of a representative volume element (RVE) is derived based on the homogenization scheme. In Section 4, the effects due to the non-conformal deformation between matrix and inclusions are described through modifying the Eshelby tensor. An approach for calculating the effective elastic modulus of RVE unit with imperfect interface is obtained. In Section 5, the estimations with this approach are compared with experimental measurements at different curing times and confining pressures. The influences of imperfect interface displacement parameters on macroscopic elastic modulus are analyzed. The conclusions are drawn in Section 6.

## 2. An Approach for Density Estimation of EPS Beads Mixed Cement-Reinforced Clay

Density is an important physical parameter of EPS beads mixed cement-reinforced clay or lightweight clay. The lightweight clay is composed of EPS beads, cement, water and marine clay and can be used as a bulk filling material in construction or geotechnical engineering. Cement-reinforced clay (called matrix later) is composed of cement, water and marine clay. EPS beads are lightweight, bulk and highly compressible. Therefore, this lightweight clay can be regarded as a two-component medium made up of EPS beads and matrix.

### 2.1. An Approach for the Density Estimation of Lightweight Clay

**Figure 2:**
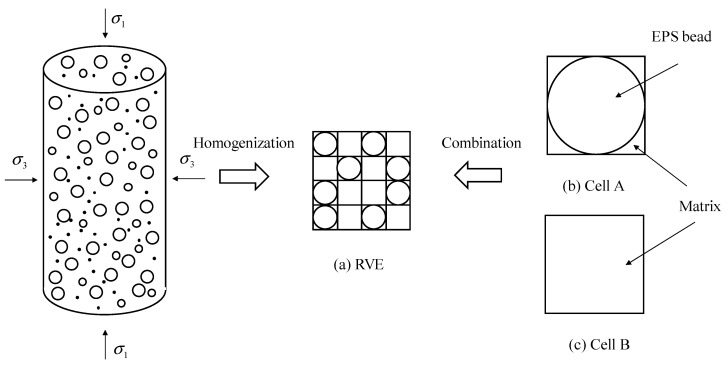
Components of lightweight bulk filling material.

The EPS beads are assumed to be evenly distributed in lightweight clay. For the convenience of homogenization process, this lightweight material is assumed to be made up of Cell A and Cell B in a RVE unit as shown in Figure 2. A simple homogenization scheme was proposed for the lightweight clay based on the concept of components [19]. Here we use cells instead of components to estimate the density of this clay as:

(1) $$\rho^{RVE}=\frac{\left(b_{s}^{\rho }-1\right)f+1}{\frac{b_{s}^{\rho }f}{\rho^{Cell\_A}}+\frac{1-f}{\rho^{Cell\_B}}}$$

where $\rho^{RVE},\rho^{Cell\_A},\rho^{Cell\_B}$ are the homogenized density of RVE unit, Cell A and Cell B, respectively. f denotes the volume ratio of EPS beads to the lightweight clay. $b_{s}^{\rho }$ is a parameter related to the density ratio of EPS beads to matrix as:

(2) $$b_{s}^{\rho }=\sqrt{\frac{\rho^{eps}}{\rho^{m}}}$$

Cell B does not contain any EPS beads, so its density is the same as that of matrix. Cell A contains only one EPS bead, thus its homogenized density is approximately expressed as:

(3) $$\rho^{Cell\_A}=\phi^{\rho }\rho^{eps}+\left(1-\phi^{\rho }\right)\rho^{m}$$

Combining Equation (3) with Equation (1) gets the density estimation of EPS beads mixed cement-reinforced clay as:

(4) $$\rho^{RVE}=\frac{\left(b_{s}^{\rho }-1\right)f+1}{\frac{b_{s}^{\rho }f}{\phi^{\rho }\rho^{eps}+\left(1-\phi^{\rho }\right)\rho^{m}}+\frac{1-f}{\rho^{m}}}$$

where $\rho^{eps},\rho^{m}$ are the density of EPS beads and matrix, respectively. $\phi^{\rho }$ is the volume ratio of EPS to Cell A and is determined by data curve fitting (in the calculation of density, the imperfect interface is regarded as a part of EPS volume).

### 2.2. Verification of Proposed Density Estimation Approach

**Figure 3:**
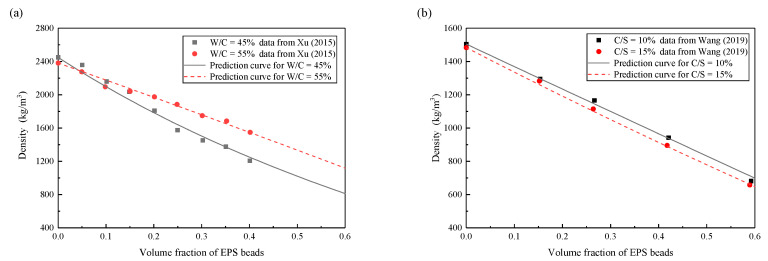
Density prediction (**a**) experimental data from Xu et al. [12]; (**b**) experimental data from Wang et al. [11].

This proposed density estimation approach is verified by two sets of experimental data in available literature. The model parameters are listed in Table 1 and the estimated densities are presented in Figure 3. These figures indicate that this approach has a good capability to fit these experimental data of density for lightweight clay.

## 3. A Homogenization Approach for Effective Modulus Estimation

The microstructure of the EPS beads mixed cement-reinforced clay is usually unknown, but the volume fraction and elastic modulus of each component can be measured. The effective elastic modulus of the RVE unit can be estimated through a simplified homogenization approach. For simplification, both stress and strain are homogenized in either Cell A or Cell B. This treatment may introduce the micro un-equilibrium at the boundaries of different cells, but it provides a good approximation for the micro-stress distribution without periodic conditions. Therefore, the stress in a RVE unit can be expressed as:

(5) $$\left[\sigma^{RVE}\right]=\left[E^{RVE}\right]\left[\varepsilon^{RVE}\right]$$

(6) $$\left[E^{RVE}\right]=\left[E^{Cell\_A}E^{Cell\_B}\right]\left[C\right]$$

where $\sigma^{RVE},\;\varepsilon^{RVE}$ are the homogenized stress and strain tensor of the REV unit, respectively. $E^{RVE}$ is the effective elastic modulus of RVE. $E^{Cell\_A},\;E^{Cell\_B}$ are the effective elastic moduli of Cell A and Cell B, respectively. A localization tensor [C] is taken as following (see [19] for details) to consider the effect of the local distribution of stress and strain:

(7) $$C=\left\{ \begin{array}{l} \frac{b_{s}}{\left(b_{s}-1\right)f+1}I,\;in\;Cell\;A\\ \frac{1}{\left(b_{s}-1\right)f+1}I,\;in\;Cell\;B \end{array}\right.$$

Substituting Equation (7) into Equation (6) obtains the effective elastic modulus of RVE as:

(8) $$E^{RVE}=\frac{\left(b_{s}-1\right)f+1}{\frac{b_{s}f}{E^{Cell\_B}}+\frac{1-f}{E^{Cell\_A}}}$$

This is an approach to estimate the effective elastic modulus of RVE unit if the volume fraction of cell B and the elastic modulus of each cell are known. I is the fourth-order unit tensor (its component is $I_{ijkl}$). $b_{s}$ is a parameter related to the interaction in the microstructure of the lightweight clay. Generally, the interaction between different cells introduces series and parallel mechanisms. These two mechanisms in a truly random material occur in the same space and at the same time. Thus, this study proposes the following formula for parameter $b_{s}$:

(9) $$b_{s}=\sqrt{\frac{E^{Cell\_A}}{E^{Cell\_B}}}$$

where $E^{Cell\_A}$ and $E^{Cell\_B}$ are the effective elastic moduli of Cell A and Cell B, respectively. $E^{Cell\_B}$ is the same as the elastic modulus of matrix.

## 4. Calculation of $E^{Cell\_A}$ Considering the Imperfect Interface

The $E^{Cell\_A}$ depends on the microstructure of Cell A. Because the EPS bead is usually much softer than matrix, the deformation of the EPS bead is much larger than that of the matrix. This may cause non-conformal deformation at the interface between EPS beads and matrix, thereby affecting the effective elastic modulus of Cell A.

### 4.1. Establishment of Interface Model

An ideal interface has continuous displacement and traction across the interface [16]:

(10) $$\Delta u_{i}=u_{i}^{S+}-u_{i}^{S-}=0$$

(11) $$\Delta \sigma_{ij}=\left[\sigma_{ij}^{S+}-\sigma_{ij}^{S-}\right]=0$$

where S represents the interface, $u_{i}^{S+},u_{i}^{S-},\sigma_{ij}^{S+},\sigma_{ij}^{S-}$ mean the displacements and tractions at the interface, respectively.

**Figure 4:**
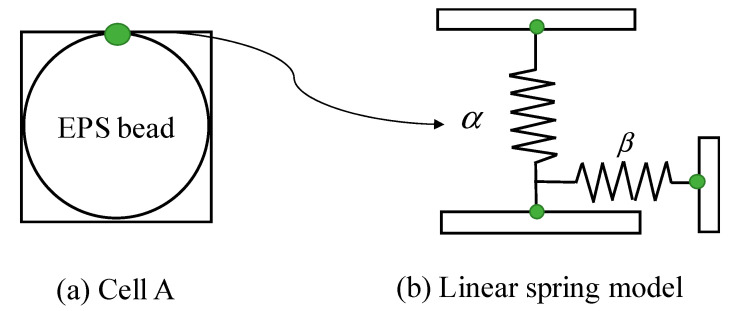
An imperfect interface simplification model.

In deformation process, the interface between EPS beads and matrix may have non-conformal deformation. This interface with non-conformal deformation is called an imperfect interface. The imperfect interface model in Cell A is shown in Figure 4, where the stress is continuous but the displacement is discontinuous:

(12) $$\Delta \sigma_{ij}n_{i}=\left[\sigma_{ij}^{S+}-\sigma_{ij}^{S-}\right]n_{i}=0$$

(13) $$\Delta u_{k}n_{l}=\eta_{klpq}\sigma_{pq}^{f}$$

For a linear spring interface, the fourth-order elastic compliance tensor $\eta_{klpq}$ is:

(14) $$\eta_{klpq}=\alpha \delta_{kl}\delta_{pq}+\left(\beta -\alpha \right)\left(\delta_{kp}\delta_{ql}+\delta_{kq}\delta_{pl}\right)$$

where $n_{j}$ is the normal direction vector of the interface S. $\eta_{klpq}=0$ corresponds to the perfectly bonded interface or ideal interface, and $\eta_{klpq}\to \infty$ represents the completely debonded interface. $\delta_{kq}$ is the Kronecker delta. α and β are the parameters of lightweight clay related to the sliding and separation of the interface, respectively:

(15) $$K_{im}=\frac{1}{3\alpha +6\beta },\;\mathrm{and}\;G_{im}=\frac{1}{4\left(\beta -\alpha \right)}$$

where $K_{im},\;G_{im}$ are the bulk and shear modulus of imperfect interface model.

### 4.2. Strain of Cell A

The deformation of Cell A is contributed from three components: the deformation of matrix, the deformation of EPS beads, and the displacement of the imperfect interface. Therefore, the homogenized strain of Cell A is:

(16) $${\bar{\varepsilon }}^{Cell\_A}=\frac{1}{V}{∭}_{V}\varepsilon dV=\phi \left(\varepsilon^{eps}+\varepsilon^{f}\right)+\left(1-\phi \right){\bar{\varepsilon }}^{m}=\phi {\bar{\varepsilon }}^{eps+f}+\left(1-\phi \right){\bar{\varepsilon }}^{m}$$

where the inclusion contains both EPS bead and interface. ${\bar{\varepsilon }}^{eps+f}$ and ${\bar{\varepsilon }}^{m}$ represent the homogenized strain of the inclusion and the matrix, respectively. ϕ denotes the volume ratio of the inclusion in Cell A.

Combining the discontinuity of displacement at the interface and the displacement of the inclusion inside Cell A gets [42]:

(17) $$u_{i}^{eps+f}=u_{i}^{eps}+u_{i}^{f}={∭}_{\Omega }\frac{\partial^{2}G_{mnkl}^{\infty }}{\partial y_{j}\partial y_{j}}E_{ijmn}^{0}\varepsilon_{kl}^{*eps}dV+{∯}_{S}\frac{\partial G_{mnkl}^{\infty }}{\partial y_{j}}E_{ijmn}^{0}\Delta u_{k}n_{l}dS$$

where $u_{i}^{eps}$ and $u_{i}^{f}$ signify the displacement of EPS bead and interface, respectively. $E_{ijmn}^{0}$ is the effective elastic stiffness of matrix, $G_{mnkl}^{\infty }$ represents the Kelvin’s fundamental solutions, and $\varepsilon_{kl}^{*eps}$ is the eigenstrain of EPS bead.

By using the Gauss theorem to convert volume integral into area integral (in the first part of Equation (17)), the strain of the inclusion in Cell A can be written as:

(18) $${\bar{\varepsilon }}_{ij}^{eps+f}=\varepsilon_{ij}^{eps}+\varepsilon_{ij}^{f}={∯}_{S}E_{ijmn}^{0}\varepsilon_{kl}^{*eps}\Gamma_{klmn}dS+{∯}_{S}E_{ijmn}^{0}\Delta u_{k}n_{l}\Gamma_{klmn}dS$$

where:

(19) $$\Gamma_{klmn}=\frac{1}{2}\left(\frac{\partial^{2}G_{mnkl}^{\infty }}{\partial y_{j}\partial y_{j}}+\frac{\partial^{2}G_{mnkl}^{\infty }}{\partial y_{i}\partial y_{i}}\right)$$

The strain of EPS bead in Cell A is expressed in terms of Eshelby tensor $S_{ijkl}$ as:

(20) $$\varepsilon_{ij}^{eps}={∯}_{S}E_{ijmn}^{0}\varepsilon_{kl}^{*eps}\Gamma_{klmn}dS=S_{ijkl}\varepsilon_{kl}^{*eps}$$

where the Eshelby tensor for linearly elastic materials ($\nu_{0}$ is the Poisson’s ratio of matrix) is [42]:

(21) $$S_{ijkl}=\frac{\left(5\nu_{0}-1\right)\delta_{ij}\delta_{kl}+\left(4-5\nu_{0}\right)\left(\delta_{ij}\delta_{kl}+\delta_{il}\delta_{jk}\right)}{15\left(1-\nu_{0}\right)}$$

In the linear-spring model, the stress caused by the eigenstrain of imperfect interface should be consistent with the stress caused by EPS bead deformation. Therefore, the stress between the interfaces can be expressed as:

(22) $$\sigma_{ij}^{f}=E_{ijkl}^{f}\varepsilon_{kl}^{*f}=E_{ijkl}^{eps}\left(\varepsilon_{kl}^{eps}-\varepsilon_{kl}^{*eps}\right)$$

where $\varepsilon_{kl}^{*f}$ and $\varepsilon_{kl}^{*eps}$ mean the eigenstrain of the interface and the EPS bead. $\varepsilon_{kl}^{eps}$ is the strain of the EPS bead. $E_{ijkl}^{f}$ and $E_{ijkl}^{eps}$ represent the elastic stiffness of the interface and EPS beads, respectively.

Simultaneous Equations (13), (18), (20) and (22), the strain of the imperfect interface $\varepsilon_{kl}^{f}$ is:

(23) $$\varepsilon_{ij}^{f}=\eta_{klpq}E_{pqst}^{eps}\left(S_{ijkl}-I_{ijkl}\right)\varepsilon_{st}^{eps}$$

Substituting Equation (23) into Equation (18) gets the strain induced by the deformations of the inclusion in Cell A as:

(24) $${\bar{\varepsilon }}_{ij}^{eps+f}=\varepsilon_{ij}^{eps}+\varepsilon_{ij}^{f}=\left[I_{ijst}+\eta_{klpq}E_{pqst}^{eps}\left(S_{ijkl}-I_{ijkl}\right)\right]\varepsilon_{st}^{eps}=S_{ijst}^{*}\varepsilon_{st}^{eps}$$

Thus, a modified Eshelby tensor $S_{ijst}^{*}$ is obtained for inclusion. The imperfect interface becomes perfect when $\eta :E^{eps}:\left(S-I\right)=0$ or η=0.

From Equation (24) to Equation (16), the homogenized strain of Cell A can be written as:

(25) $${\bar{\varepsilon }}^{Cell\_A}=\phi S^{*}\varepsilon^{eps}+\left(1-\phi \right){\bar{\varepsilon }}^{m}$$

According to the Eshelby equivalence equation, the stress caused by the effect of the EPS bead is the same as that of the matrix. Hence, this relationship is:

(26) $$E^{eps}\left({\bar{\varepsilon }}^{m}+\varepsilon^{eps}\right)=E^{0}\left({\bar{\varepsilon }}^{m}+\varepsilon^{eps}-\varepsilon^{*eps}\right)$$

where $E^{eps},E^{0}$ are the effective stiffness of the EPS bead and matrix, respectively.

Substituting Equation (26) into Equation (25) obtains the homogenized strain of Cell A as:

(27) $${\bar{\varepsilon }}^{Cell\_A}=\left\{\phi \left[I+\eta E^{eps}\left(S-I\right)\right]S\left(E^{0}-E^{eps}\right){\left[\left(E^{eps}-E^{0}\right)S+E^{0}\right]}^{-1}+\left(1-\phi \right)I\right\}{\bar{\varepsilon }}^{m}$$

### 4.3. Effective Elastic Modulus of RVE Unit

When a body is subjected to the traction boundary condition with a constant stress tensor, the average stress over the entire body is the same as the boundary pressure, regardless of stress complexity within the domain [43]. Therefore, the traction boundary condition is:

(28) $${\sigma_{ij}n_{j}|}_{s}={\bar{\sigma }}_{ij}n_{j}$$

Through the homogenization approach, the average stress of Cell A can be obtained as:

(29) $$\langle \sigma_{ij}\rangle =\frac{1}{V}{∭}_{V}\sigma_{ij}dV_{x}=\frac{1}{V}\left({∭}_{V_{eps}}\sigma_{ij}dV_{x}+{∭}_{V_{m}}\sigma_{ij}dV_{x}\right)=\phi {\bar{\sigma }}_{ij}^{eps}+\left(1-\phi \right){\bar{\sigma }}_{ij}^{m}$$

where $V_{eps},V_{m}$ are the volume of EPS bead and matrix, respectively. ${\bar{\sigma }}_{ij}^{eps},{\bar{\sigma }}_{ij}^{m}$ are the average stress of inclusion and matrix, respectively.

According to Equation (20) and Equation (26), the average stress ${\bar{\sigma }}_{ij}^{eps}$ caused by the EPS bead and discontinuous displacement at the interface can be expressed as:

(30) $${\bar{\sigma }}_{ij}^{eps}=E^{eps}:\varepsilon^{eps}=E^{eps}:S:\left(E^{0}-E^{eps}\right):{\left[\left(E^{eps}-E^{0}\right):S+E^{0}\right]}^{-1}:{\bar{\varepsilon }}^{m}$$

Substituting the Equation (30) into Equation (29) obtains the homogenized stress of Cell A as:

(31) $${\bar{\sigma }}^{Cell\_A}=\left\{\phi E^{eps}S\left(E^{0}-E^{eps}\right){\left[\left(E^{eps}-E^{0}\right)S+E^{0}\right]}^{-1}+\left(1-\phi \right)E^{0}\right\}{\bar{\varepsilon }}^{m}$$

According to Equation (27) and Equation (31), the effective modulus of Cell A is obtained as:

(32) $$E^{Cell\_A}=\left\{\begin{array}{l} \phi {\left[\left(E^{eps}-E^{0}\right)S+E^{0}\right]}^{-1}S\\ \left(E^{0}-E^{eps}\right)E^{eps}+\left(1-\phi \right)E^{0} \end{array}\right\}{\left\{\begin{array}{l} \phi {\left[\left(E^{eps}-E^{0}\right)S+E^{0}\right]}^{-1}S\left(E^{0}-E^{eps}\right)\\ \left[I+\eta :E^{eps}:\left(S-I\right)\right]+\left(1-\phi \right)I \end{array}\right\}}^{-1}$$

Therefore, the effective modulus of RVE unit is obtained from Equation (32) and Equation (8). This effective modulus is the result after combining the modified Eshelby tensor with imperfect interfaces and the multi-scale homogenization approach for a RVE unit. In this estimation approach, the parameters α and β are for imperfect interface and may lead to a distinct change in the effective modulus of the lightweight bulk filling material.

## 5. Validation of the Proposed Homogenization Approach for Effective Modulus

This estimation approach is firstly verified by our experimental data. The effects of curing time, confining pressure and imperfect interface parameters are then investigated. For the lightweight clay [11], the basic parameters are listed in Table 2.

### 5.1. Effect of Curing Time

**Figure 5:**
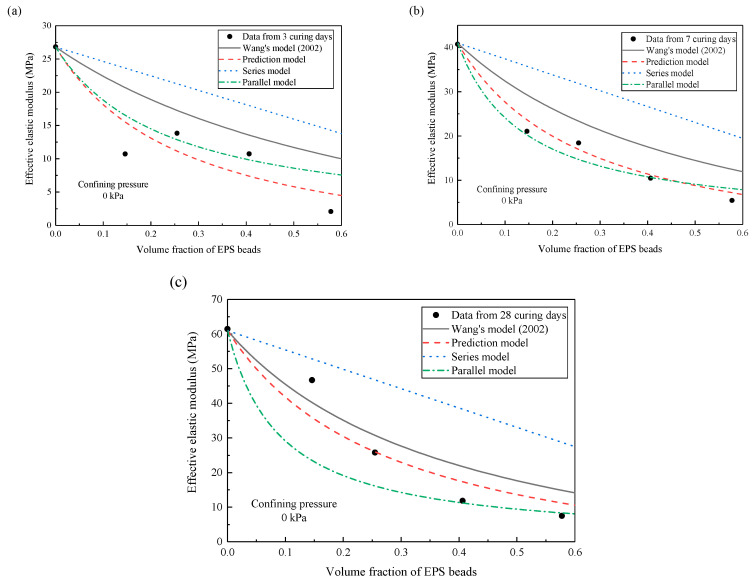
Comparison of effective elastic modulus predicted by models with experimental data from Wang et al. [11]. (**a**) 3 curing days. (**b**) 7 curing days. (**c**) 28 curing days.

Four estimation models are compared with the experimental effective elastic moduli of lightweight clay at the curing time of 3 days, 7 days and 28 days [11]. The first estimation model is the series model, where the effective elastic modulus of the composite material is calculated by the weighted average of the elastic moduli of matrix and inclusions. The weight is usually taken as volume fraction. The second one is the parallel model, where the effective elastic modulus of composite material is obtained through the weighted average of the reciprocal of the elastic moduli of matrix and inclusions. Wang et al. [19] proposed an estimation model based on homogenization approach (called Wang’s model (2002)), but this model did not consider the effects of imperfect interfaces. Figure 5 compares the effective elastic moduli predicted by these four estimation models. When the curing time is 3 days, the prediction results of series-parallel model and Wang’s model are larger than the experimental data. Our prediction model is in good agreement with the experimental data. The series and parallel models can roughly estimate the effective elastic modulus of composite materials when the volume fraction of inclusions is less than 40% (as shown in Figure 5b). However, the results predicted by the series and parallel models are significantly higher than the experimental data when the volume fraction of inclusions is more than 40%. As the volume fraction of inclusions increases, the imperfect interface increases and has a significant influence on the elastic modulus of the composite material. Wang’s model (2002) is the average of the series and parallel models, thus its prediction result is always between the prediction results of the series model and the parallel model. Therefore, the approach proposed in this study can predict the effective modulus more accurately due to the consideration of imperfect interfaces.

The elastic modulus of matrix increases with curing time. At the zero confining pressure, the elastic modulus of matrix is 26.8 MPa at 3 days, 40.7 MPa at 7 days, and 61.4 MPa at 28 days. This indicates that the strength of matrix has not been completely stabilized within the short curing time. Therefore, the deformation of matrix is greater at the earlier curing time. The influence of the imperfect interface between the matrix and the inclusions is significant as the volume fraction of the inclusions increases.

### 5.2. Effect of Confining Pressure

**Figure 6:**
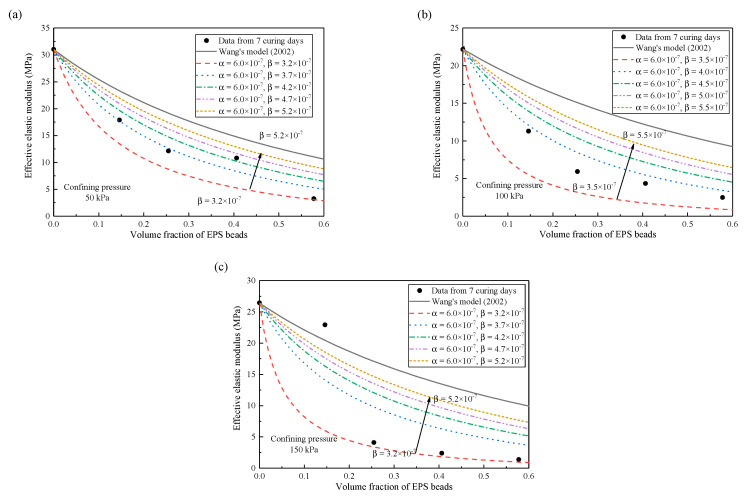
Effects of imperfect interface parameter *β* on effective elastic modulus under different confining pressures. (**a**) 50 kPa. (**b**) 100 kPa. (**c**) 150 kPa.

**Figure 7:**
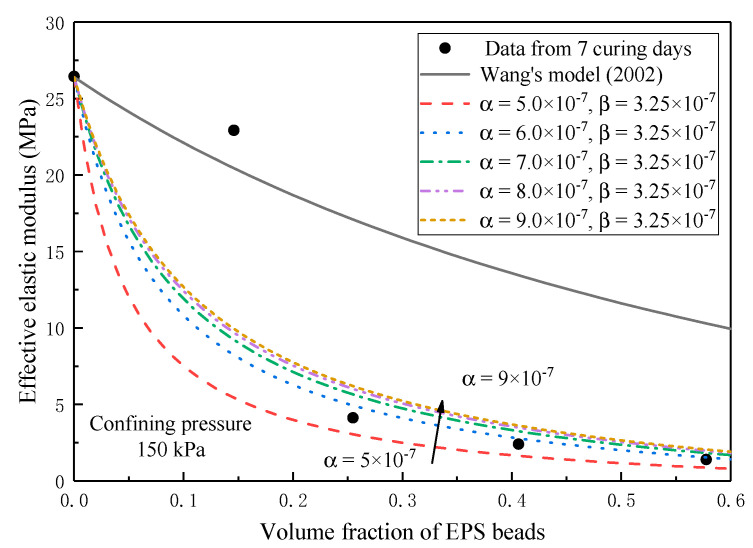
Effect of imperfect interface parameter *α* on effective elastic modulus under confining pressure of 150 kPa.

Confining pressure affects the compressibility of matrix and EPS inclusions, thereby affecting the imperfect interface. The experimental data at 7-days curing time are taken for further analysis. The results are presented in Figure 6 for *β* effect and in Figure 7 for *α* effect. At this time, the elastic modulus of matrix is 31 MPa, 22.2 MPa, and 26.4 MPa when the confining pressure is 50 kPa, 100 kPa, and 150 kPa, respectively. In order to further explore the influence of imperfect interface on the elastic modulus of lightweight clay at different volume fractions, Figure 6 presents the effect of interface parameter *β* on the effective elastic modulus. The prediction curve gradually approaches to the curve of the homogenization approach as the *β* increases. The model with *β =* 3.7 × 10^−7^ can well predict the experimental data when the confining pressure is 50 kPa. When the confining pressure is 100 kPa, the model with *β =* 3.8 × 10^−7^ can well predict the experimental data. *β =* 3.25 × 10^−7^ is the best one to predict the experimental data when the confining pressure is 150 kPa. Further, Figure 7 shows that the curvature of the predicted curve decreases with the increase of the parameter *α*, which is the same change as the parameter *β*, but the change of *α* is not as salient as that of the parameter *β*.

Generally, the prediction curve is approaching to the curve of homogenization approach as the parameters of imperfect interface increase. This implies that larger interface parameter has less obvious non-conformal deformation at the interface. Under different confining pressures, the parameter *β* has a greater impact on the change of prediction curve than the parameter *α*. The prediction curve is far from the curve of the homogenization approach when the confining pressure is larger. This means that confining pressure has a significant effect on imperfect interface.

### 5.3. Variations of Interface Parameters with Curing Time and Confining Pressure

**Figure 8:**
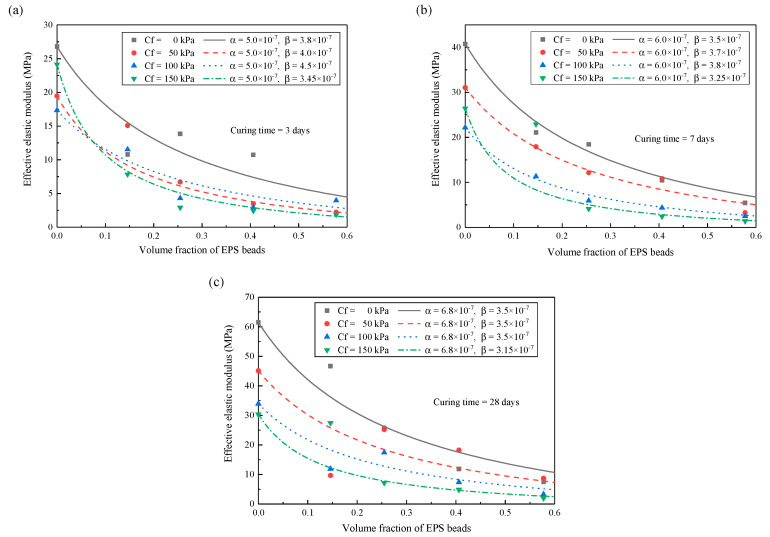
Variation of effective elastic modulus with volume fraction at different curing times and confining pressures (**a**) 3-days curing time; (**b**) 7-days curing time; (**c**) 28-days curing time.

The variations of interface parameters with curing time and confining pressure are investigated here. Figure 8 presents the relationship between curing time and imperfect interface parameters. Figure 8a presents the effective elastic modulus of lightweight clay with the volume fractions of inclusions when the curing time is 3 days. With the increase of confining pressure, the parameter α is constant, while the parameter *β* increases first and then decreases. A similar phenomenon is observed when the curing time is 7 days (see Figure 8b). Figure 8c indicates that the material is fully cured at the 28-days curing time. At this time, the elastic modulus of the matrix decreases with the increase of confining pressure. Under the same volume fraction, greater confining pressure makes the lightweight clay have lower elastic modulus. The *β* changes differently at 3-days and 7-days curing time from the 28-days. At the curing time of 28 days, the *β* decreases gradually as the confining pressure increases.

**Figure 9:**
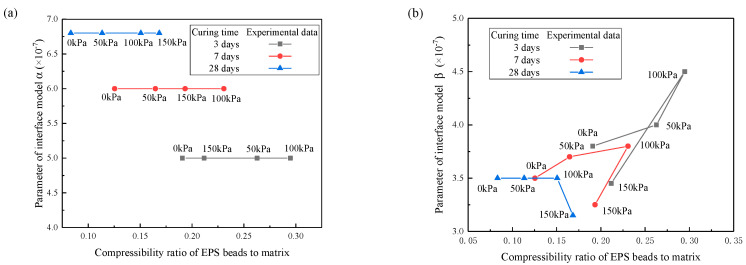
The compressibility ratio at different curing times (**a**) parameter of interface model *α*; (**b**) parameter of interface model *β*.

The relationship between the parameters of imperfect interface and the compressibility ratio is presented in Figure 9. The compressibility ratio is defined as the ratio of the elastic modulus of EPS beads to the elastic modulus of matrix. This figure indicates that the compressibility ratio decreases with the curing time at the same confining pressure. This is because the elastic modulus of matrix increases with curing time before 28 days. In a shorter curing time, the compressibility ratio increases first and then decreases as the confining pressure increases. At the 28-days curing time, the compressibility ratio increases with the increase of confining pressures. When the confining pressure is constant, the compressibility ratio of lightweight clay decreases with the increases of curing time. Figure 9a indicates that the parameter *α* does not change with confining pressure if the curing time is the same. Under the same confining pressure, the parameter *α* increases as the compressibility ratio decreases. Figure 9b indicates the change of the parameter *β*. Under the same confining pressure, longer curing time corresponds to smaller parameter *β*. The parameter *β* decreases as the compressibility ratio decreases.

## 6. Conclusions

This study proposed an approach to estimate the density and effective elastic modulus of a lightweight bulk filling material. The lightweight clay is made up of expanded polystyrene (EPS) beads and cement-reinforced clay. The physical and mechanical properties of EPS beads and cement-reinforced clay are different and may induce non-conformal deformation at the interface between different components under compression. This non-conformal deformation is described by the imperfect interface model and introduced into the modified Eshelby tensor. Finally, an approach to estimate the density and effective elastic modulus of the lightweight filling material is developed through a homogenization approach. This estimation approach was verified by the experimental data at different confining pressures and different curing times. The effects of interface parameters were also explored. Based on these investigations, following conclusions can be drawn.

Firstly, this estimation approach is the extension of homogenization approach through a composite RVE unit. This extension can well describe the microstructure of the EPS mixed cement-reinforced clay. The non-conformal deformation at the imperfect interface is included through a modification of the Eshelby tensor, and the effective strain and effective stress in Cell A are derived.

Secondly, the effective elastic modulus of lightweight clay decreases with the increase of volume fraction of inclusions. This change is modified by curing time and confining pressure. The imperfect interface parameters *α* and *β* vary with the curing time and confining pressure. At the same curing time, the parameter *α* is almost constant regardless of confining pressure but the parameter *β* changes with confining pressure. As the curing time increases, the parameter *α* increases while the parameter *β* decreases. This indicates that both curing time and confining pressure have a great impact on the non-conformal deformation at the imperfect interface.

Finally, a compressibility ratio is an important parameter to affect the non-conformal deformation of EPS beads and matrix in lightweight clay. It can comprehensively explain the effects of curing time, confining pressure, and interface. When the volume fraction of inclusions increases, the deformation at the interface is more non-conformal due to the large difference in elastic modulus between the matrix and the inclusions. When the compressibility ratio is low, the difference between the elastic moduli of the EPS beads and the matrix becomes large and the effect of imperfect interface becomes salient. This greatly damages the effective elastic modulus of the lightweight filling material.

## Figures and Tables

**Table 1:** Components of lightweight clay.

Cement Content	$b_{s}$	$\phi^{\rho }$	$\rho^{eps}(\mathrm{kg}/m^{3})$	$\rho^{m}(\mathrm{kg}/m^{3})$	Experimental Data Sources
C/S = 10%	0.1153	0.8985	20	1504.4	Wang et al. [11]
C/S = 15%	0.1161	0.9090	20	1483.4	Wang et al. [11]
W/C = 45%	0.0904	0.9518	20	2449.2	Xu et al. [12]
W/C = 55%	0.0916	0.9110	20	2381.4	Xu et al. [12]

C/S denotes the mass ratio of cement to clay; W/C is the mass ratio of water to cement.

**Table 2:** Mechanical properties of components and parameters of imperfect interface model.

Curing Time (days)	Confining Pressure (kPa)	ϕ	Interface Parameters		EPS Beads		Matrix	
			α $\left(\times 10^{-7}\right)$	β $\left(\times 10^{-7}\right)$	Elastic Modulus (MPa)	Poisson’s Ratio	Elastic Modulus (MPa)	Poisson’s Ratio
3	0	$\frac{\pi }{6}$	5.0	3.8	5.112	0.4	26.8	0.3
7	0	$\frac{\pi }{6}$	6.0	3.5	5.112	0.4	40.7	0.3
28	0	$\frac{\pi }{6}$	6.8	3.5	5.112	0.4	61.4	0.3
7	50	$\frac{\pi }{6}$	6.0	3.2~5.2	5.112	0.4	31.0	0.3
7	100	$\frac{\pi }{6}$	6.0	3.5~5.5	5.112	0.4	22.2	0.3
7	150	$\frac{\pi }{6}$	6.0	3.2~5.2	5.112	0.4	26.4	0.3
7	150	$\frac{\pi }{6}$	5.0~9.0	3.25	5.112	0.4	26.4	0.3

Experimental data from Wang et al. [11].

## Data Availability

The raw/processed data required to reproduce these findings cannot be shared at this time due to technical or time limitations.

## References

[1] Solak A.M., Tenza-Abril A.J., Baeza-Brotons F., Benavente D. (2019). Proposing a New Method Based on Image Analysis to Estimate the Segregation Index of Lightweight Aggregate Concretes. Materials.

[2] Dixit A., Pang S.D., Kang S.-H., Moon J. (2019). Lightweight structural cement composites with expanded polystyrene (EPS) for enhanced thermal insulation. Cem. Concr. Compos..

[3] Gao H., Bu C., Wang Z., Shen Y., Chen G. (2017). Dynamic characteristics of expanded polystyrene composite soil under traffic loadings considering initial consolidation state. Soil Dyn. Earthq. Eng..

[4] Herki B., Khatib J. (2016). Valorisation of waste expanded polystyrene in concrete using a novel recycling technique. Eur. J. Environ. Civ. Eng..

[5] Wang F., Miao L. (2009). A proposed lightweight fill for embankments using cement-treated Yangzi River sand and expanded polystyrene (EPS) beads. Bull. Eng. Geol. Environ..

[6] Miled K., Sab K., Le Roy R. (2007). Particle size effect on EPS lightweight concrete compressive strength: Experimental investigation and modelling. Mech. Mater..

[7] Gao H., Chen Y., Liu H., Liu J., Chu J. (2012). Creep behavior of EPS composite soil. Sci. China-Technol. Sci..

[8] Nguyen-Sy T., Tran-Le A., Nguyen-Thoi T., Langlet T. (2017). A multi-scale homogenization approach for the effective thermal conductivity of dry lime–hemp concrete. J. Build. Perform. Simul..

[9] Khosravani M.R., Nasiri S., Anders D., Weinberg K. (2019). Prediction of dynamic properties of ultra-high performance concrete by an artificial intelligence approach. Adv. Eng. Softw..

[10] Ren L., Yu X., He Y., Wang K., Yao H. (2020). Numerical investigation of lateral inertia effect in dynamic impact testing of UHPC using a Split-Hopkinson pressure bar. Constr. Build. Mater..

[11] Wang J., Hu B., Soon J.H. (2019). Physical and Mechanical Properties of a Bulk Lightweight Concrete with Expanded Polystyrene (EPS) Beads and Soft Marine Clay. Materials.

[12] Xu J., Chu H., Xu Y., Li Y., Jiang L. (2015). Prediction of compressive strength and elastic modulus of expanded polystyrene lightweight concrete. Mag. Concr. Res..

[13] Brovelli A., Cassiani G. (2010). A combination of the Hashin-Shtrikman bounds aimed at modelling electrical conductivity and permittivity of variably saturated porous media. Geophys. J. Int..

[14] Festugato L., Da Silva A.P., Diambra A., Consoli N.C., Ibraim E. (2018). Modelling tensile/compressive strength ratio of fibre reinforced cemented soils. Geotext. Geomembr..

[15] Liu B., Wang H., Qin Q.H. (2018). Modelling and Characterization of Effective Thermal Conductivity of Single Hollow Glass Microsphere and Its Powder. Materials.

[16] Wang J., Luo J. (2017). Micromechanical study on the effective elastic moduli of polymer-bonded explosives with imperfect interfaces. J. Energ. Mater..

[17] Nguyen L., Fatahi B., Khabbaz H. (2016). Predicting the Behaviour of Fibre Reinforced Cement Treated Clay. Procedia Eng..

[18] Nguyen L., Fatahi B. (2016). Behaviour of clay treated with cement & fibre while capturing cementation degradation and fibre failure—C3F Model. Int. J. Plast..

[19] Wang J., Leung C., Ichikawa Y. (2002). A simplified homogenisation method for composite soils. Comput. Geotech..

[20] Chen Y., Zhou S., Hu R., Zhou C.-B. (2015). A homogenization-based model for estimating effective thermal conductivity of unsaturated compacted bentonites. Int. J. Heat Mass Transf..

[21] Song Z., Peng X., Tang S., Fu T. (2020). A homogenization scheme for elastoplastic composites using concept of Mori-Tanaka method and average deformation power rate density. Int. J. Plast..

[22] Eshelby J.D., Jayaratne O.W., Mason B.J. (1957). The determination of the elastic field of an ellipsoidal inclusion, and related problems. Proc. R. Soc. Lond. Ser. A Math. Phys. Sci..

[23] Kanoute P., Boso D.P., Chaboche J.L., Schrefler B.A. (2009). Multiscale Methods for Composites: A Review. Arch. Comput. Methods Eng..

[24] Peng X., Tang S., Hu N., Han J. (2016). Determination of the Eshelby tensor in mean-field schemes for evaluation of mechanical properties of elastoplastic composites. Int. J. Plast..

[25] Mori T., Tanaka K. (1973). Average stress in matrix and average elastic energy of materials with misfitting inclusions. Acta Metall..

[26] Klusemann B., Böhm H.J., Svendsen B. (2012). Homogenization methods for multi-phase elastic composites with non-elliptical reinforcements: Comparisons and benchmarks. Eur. J. Mech. A Solids.

[27] Feyel F., Chaboche J.L. (2000). FE2 multiscale approach for modelling the elastoviscoplastic behaviour of long fibre SiC/Ti composite materials. Comput. Methods Appl. Mech. Eng..

[28] Benveniste Y. (1987). A new approach to the application of Mori-Tanaka’s theory in composite materials. Mech. Mater..

[29] Tan H., Huang Y., Liu C., Geubelle P. (2005). The Mori–Tanaka method for composite materials with nonlinear interface debonding. Int. J. Plast..

[30] Benveniste Y., Miloh T. (2001). Imperfect soft and stiff interfaces in two-dimensional elasticity. Mech. Mater..

[31] Hashin Z. (2001). Thin interphase/imperfect interface in conduction. J. Appl. Phys..

[32] Hashin Z. (2002). Thin interphase/imperfect interface in elasticity with application to coated fiber composites. J. Mech. Phys. Solids.

[33] Hua Y., Gu L., Premaraj S., Zhang X. (2015). Role of Interphase in the Mechanical Behavior of Silica/Epoxy Resin Nanocomposites. Materials.

[34] Shodja H.M., Hashemian B. (2019). Variational bounds and overall shear modulus of nano-composites with interfacial damage in anti-plane couple stress elasticity. Int. J. Damage Mech..

[35] Lee J., Kim J.-S., Cho M. (2020). An asymptotic method-based composite plate model considering imperfect interfaces. Int. J. Solids Struct..

[36] Tian W., Fu M.W., Qi L., Ruan H. (2020). Micro-mechanical model for the effective thermal conductivity of the multi-oriented inclusions reinforced composites with imperfect interfaces. Int. J. Heat Mass Transf..

[37] Dumont S., Lebon F.C., Raffa M.L., Rizzoni R., Welemane H. (2016). Multiscale Modeling of Imperfect Interfaces and Applications. Comput. Methods Appl. Sci..

[38] Wang G., Tu W., Chen Q. (2019). Homogenization and localization of imperfectly bonded periodic fiber-reinforced composites. Mech. Mater..

[39] Miled K., Limam O. (2016). Effective thermal conductivity of foam concretes: Homogenization schemes vs experimental data and FEM simulations. Mech. Res. Commun..

[40] Chen E.L., Ang W.T. (2014). Green’s functions and boundary element analysis for bimaterials with soft and stiff planar interfaces under plane elastostatic deformations. Eng. Anal. Bound. Elem..

[41] Kuo H.-Y. (2013). Effective property of multiferroic fibrous composites with imperfect interfaces. Smart Mater. Struct..

[42] Yanase K., Ju J.W. (2011). Effective Elastic Moduli of Spherical Particle Reinforced Composites Containing Imperfect Interfaces. Int. J. Damage Mech..

[43] Qu J., Cherkaoui M. (2006). Fundamentals of Micromechanics of Solids.

